# Copy number variability in Parkinson’s disease: assembling the puzzle through a *systems biology* approach

**DOI:** 10.1007/s00439-016-1749-4

**Published:** 2016-11-28

**Authors:** Valentina La Cognata, Giovanna Morello, Velia D’Agata, Sebastiano Cavallaro

**Affiliations:** 1 Institute of Neurological Sciences, National Research Council, Catania, Italy; 2 Section of Human Anatomy and Histology, Department of Biomedical and Biotechnological Sciences, University of Catania, Catania, Italy

## Abstract

Parkinson’s disease (PD), the second most common progressive neurodegenerative disorder of aging, was long believed to be a non-genetic sporadic origin syndrome. The proof that several genetic loci are responsible for rare Mendelian forms has represented a revolutionary breakthrough, enabling to reveal molecular mechanisms underlying this debilitating still incurable condition. While single nucleotide polymorphisms (SNPs) and small indels constitute the most commonly investigated DNA variations accounting for only a limited number of PD cases, larger genomic molecular rearrangements have emerged as significant PD-causing mutations, including submicroscopic Copy Number Variations (CNVs). CNVs constitute a prevalent source of genomic variations and substantially participate in each individual’s genomic makeup and phenotypic outcome. However, the majority of genetic studies have focused their attention on single candidate-gene mutations or on common variants reaching a significant statistical level of acceptance. This gene-centric approach is insufficient to uncover the genetic background of polygenic multifactorial disorders like PD, and potentially masks rare individual CNVs that all together might contribute to disease development or progression. In this review, we will discuss literature and bioinformatic data describing the involvement of CNVs on PD pathobiology. We will analyze the most frequent copy number changes in familiar PD genes and provide a “*systems biology*” overview of rare individual rearrangements that could functionally act on commonly deregulated molecular pathways. Assessing the global genome-wide burden of CNVs in PD patients may reveal new disease-related molecular mechanisms, and open the window to a new possible genetic scenario in the unsolved PD puzzle.

**Electronic supplementary material:**

The online version of this article (doi:10.1007/s00439-016-1749-4) contains supplementary material, which is available to authorized users.

## Introduction

**Fig. 1:**
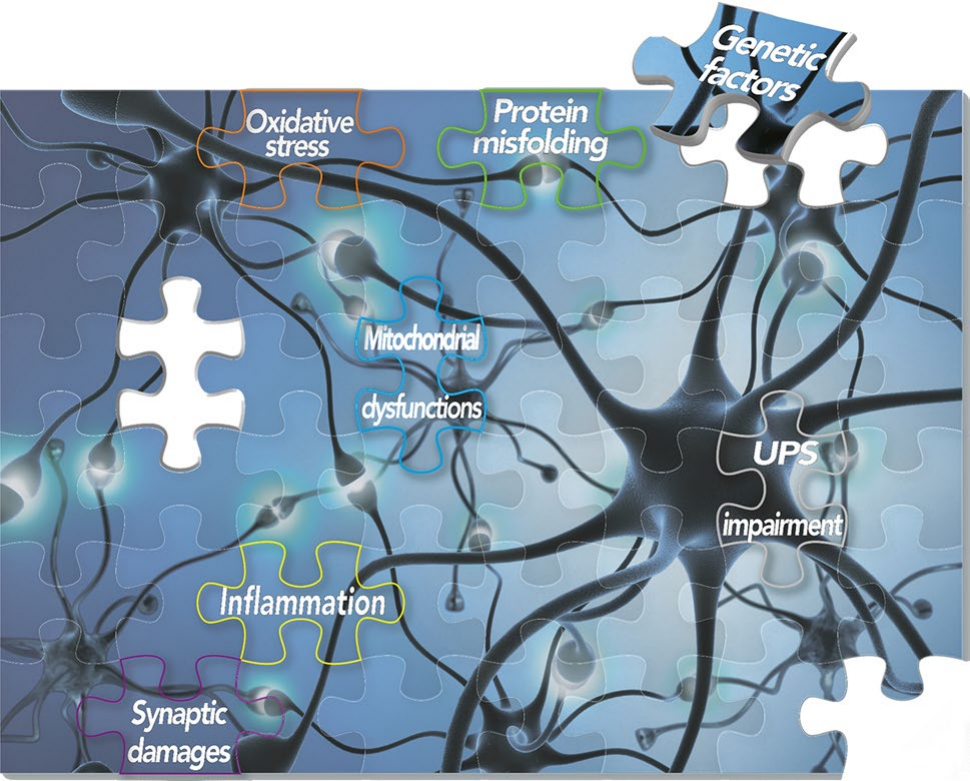
Schematic representation of molecular elements and common altered pathways underlying the complex PD puzzle

Parkinson’s disease (PD) is a progressive debilitating movement disorder, affecting approximately 1% of the population over 65 (Moore et al. 2005). The characteristic major motor symptoms derive from the profound and selective loss of dopaminergic neurons from *substantia nigra pars compacta*, coupled with an accumulation of round cytoplasmic inclusions (Lewy bodies) and dystrophic neurites (Lewy neurites) in surviving neurons (Moore et al. 2005). In more advanced stages, patients can also develop a range of nonmotor symptoms, including rapid eye movement, sleep behavior disorder, constipation, depression and cognitive decline. Treatments aimed at compensating dopamine deficit (such as levodopa and deep brain stimulation) can alleviate the motor symptoms but finally are not effective to halt or slow down disease progression (Toft and Ross 2010). Despite the molecular mechanisms underlying PD are still far from being understood, the progressive deterioration of vulnerable dopaminergic neurons seems to arise from several cellular disturbances including protein misfolding and aggregation (Michel et al. 2016), synaptic damages, apoptosis, mitochondrial dysfunctions (Winklhofer and Haass 2010), oxidative stress (Dias et al. 2013), impairment of the Ubiquitin/Proteasome System (UPS) (Betarbet et al. 2005) and neuroinflammation (Wang et al. 2015) (Fig. 1).

PD was for a long time believed to be a typical non-genetic disorder. When in 1997 Polymeropoulos and colleagues reported the first *SNCA* pathogenic mutation in the Italian Contursi kindred (Polymeropoulos et al. 1997), they revolutionized this view opening the way to new interesting perspectives about the genetic contribution to this still incurable condition (Moore et al. 2005). From that moment, an increasing number of genetic loci and numerous risk factors have been discovered (Lewis and Cookson 2012; Bonifati 2014), starting from the familiar genes responsible for the Mendelian inherited forms, such as the autosomal dominant genes (*SNCA*, *LRRK2*, *VPS35*, *GBA*), the typical recessive (*PARK2*, *PINK1*, *PARK7*) and the atypical recessive ones (*ATP13A2*, *PLA2G6*, *FBXO7*) (Klein and Westenberger 2012). Despite the existence of these rare monogenic forms, it is now clear that PD is a genetically heterogeneous and most likely complex disorder, often complicated by incomplete penetrant traits and variable expressivity. The list of candidate genes is continuously updated (Bonifati 2014; Lubbe and Morris 2014; Trinh and Farrer 2013), mainly thanks to the massive advancement in genomic biotechnologies that have allowed to detect hundreds of pathogenic or susceptibility variants at the single nucleotide polymorphism (SNP) level. However, a lot of work still has to be done to identify additional sources of missing heritability or to assign a precise causal mechanism to the growing number of discovered loci (Gamazon and Stranger 2015).

While SNPs and small indels constitute the most commonly investigated DNA variations, submicroscopic chromosomal rearrangements, also known as Copy Number Variations (CNVs), are emerging as crucial players in the individual’s genomic architecture and in modeling complex human diseases, including PD. However, the majority of CNV association studies have been conducted using the traditional candidate-gene approach that, although provides valuable information on common variants, is inadequate to completely dissect the genetic background of polygenic multifactorial disorders like PD. The search for single-gene mutations has to be changed, turning into the need to assess the collective effect of common and rare variants that together may converge on PD pathology. In this context, the “*systems biology*” approach represents a worthwhile instrument to analyze complex biological systems, moving beyond the conventional gene-centric scheme, and finally generating a more defined molecular picture of PD.

Herein, we will review the most common CNV-altered genes and detail the current knowledge about their pathogenic or susceptibility impact on PD pathobiology. Moreover, we will collect the set of rare individual CNVs reported so far in PD patients and analyze them by a “*systems biology*” approach. This new perspective reveals these private CNVs cluster in common deregulated biological processes that could contribute to disease onset or progression, and opens the window to a new possible genetic scenario in the unsolved PD puzzle.

## Copy number variations: a prevalent source of genomic variations

The DNA sequence of human genome is constantly changing and this process allows humans to evolve and adapt. The scientific community has long been aware of genetic variations of extreme size (i.e., cytogenetically recognizable elements and SNPs) (Zarrei et al. 2015). However, about 10 years ago, scientists began to recognize abundant variations of an intermediate size class known as structural variations. Within this class, copy number variations represent the largest component by far. CNVs are defined as genomic segments showing copy number variability among individuals compared to a reference genome. The size of CNVs ranges from 50 bp to several Mb, with a significant drop of variant numbers in 50 bp to 1 kb range (MacDonald et al. 2014). These structural variants can include either a single gene or a contiguous set of genes, encompassing more polymorphic base pairs than SNPs and finally resulting in an altered DNA diploid status (i.e., gain or loss of genomic region).

**Fig. 2:**
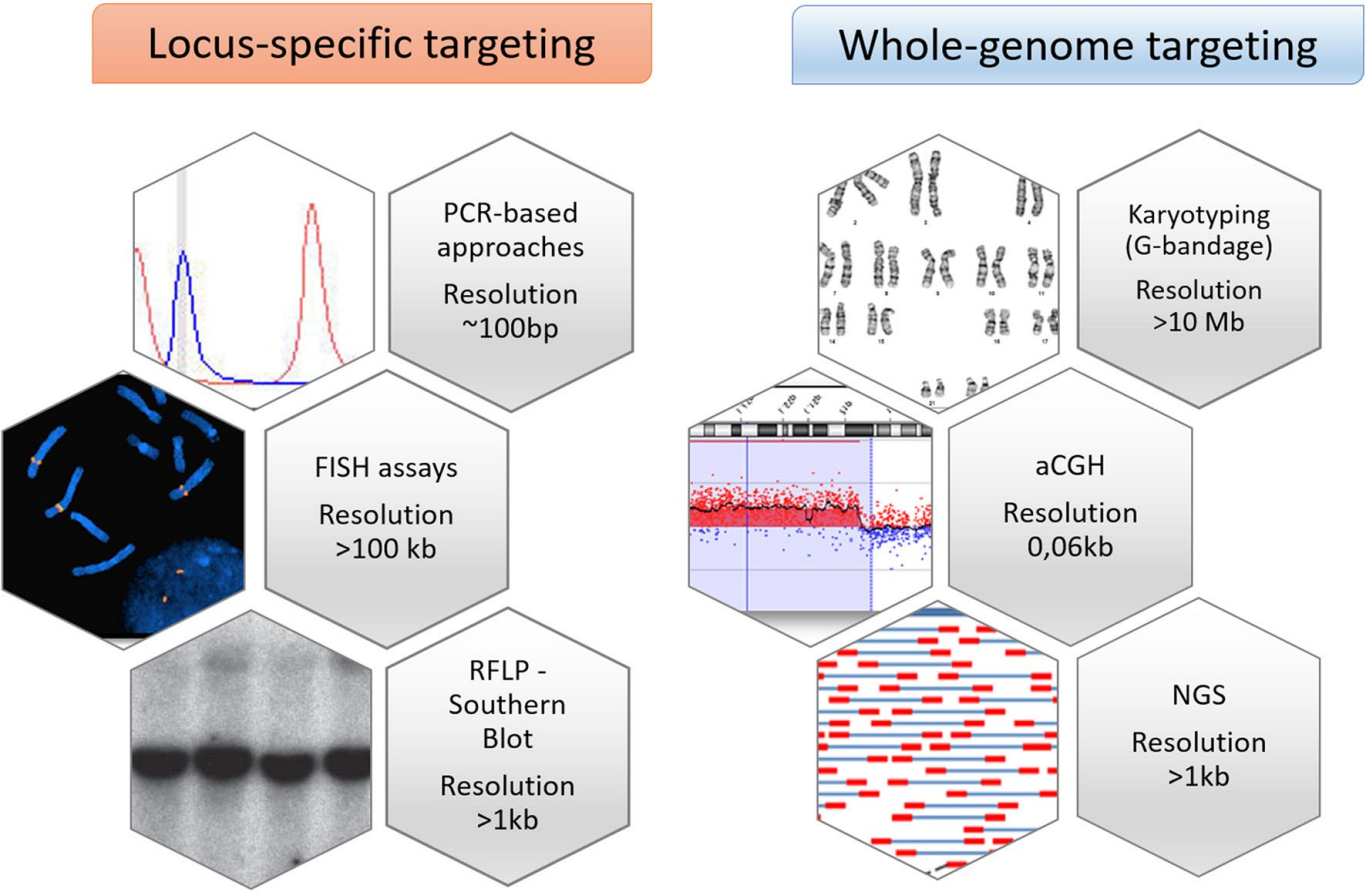
CNVs can be measured by a spectrum of laboratory methods targeting specific locations on chromosomes (locus-specific levels), or the whole genome (genome-wide level). These numerous methodologies are characterized by different levels of resolutions. The locus-specific techniques encompass (1) PCR-based strategies, such as quantitative real-time PCR (qPCR), Multiplex Ligand Probe Amplification (MLPA) or multiplex amplifiable probe hybridization (MAPH); (2) the Fluorescence in situ Hybridization (FISH) assays and (3) the RFLP (restriction fragment length polymorphism—Southern blot analysis. The whole-genome methodologies include (1) the classical chromosomal G-bandage (karyotyping); (2) the aCGH (Comparative Genomic Hybridization array) platforms and (3) the NGS (Next-Generation sequencing) technology. These two latter are increasingly replacing both the classical detections methods and the locus-specific techniques

Depending on their size, CNVs can be measured by a multitude of laboratory testing methods, either targeting the whole genome (genome-wide level) or restricted to certain locations on chromosomes (locus-specific levels) (Fig. 2) (Cantsilieris et al. 2013). While targeted approaches such as FISH or quantitative PCR-based strategies have been long used in the past, the most advanced screenings rely on whole-genome applications, such as array Comparative Genomic Hybridization or Next-Generation Sequencing experiments. Both these biotechnologies have dramatically improved and catalyzed the detection and characterization of multiple CNVs, offering the simultaneous testing of thousands of loci with high reproducibility, high resolution, and scalability for complete mapping of imbalances (Carter et al. 2002; Shaw-Smith et al. 2004; Iafrate et al. 2004; Inazawa et al. 2004; Ishkanian et al. 2004). However, these whole-genome strategies still need post-experimental validations and, therefore, a gold-standard analysis for CNVs has not been defined yet.

**Fig. 3:**
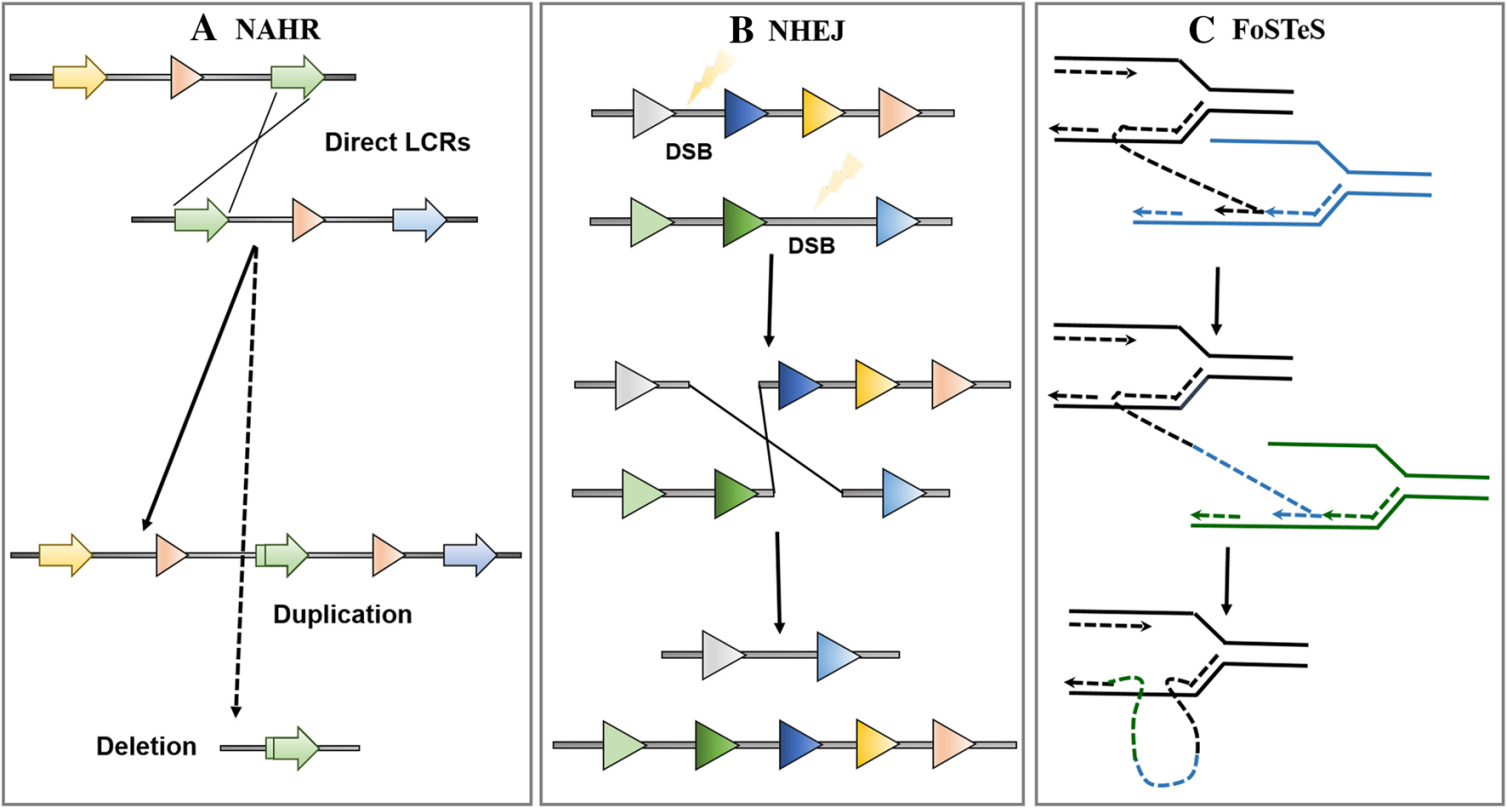
Schematic illustration of the three most common events causing genomic rearrangements. **a** NAHR generates CNVs when genomic segments with high sequence similarity (direct low-copy repeats sequences, *green arrows*) recombine. This recombination can generate a duplication of the similar locus (*red arrow*) on one chromosome, while removing the copy from the other. **b** Double-stranded breaks (DBS) in DNA sequence recruit NHEJ-associated proteins to repair and ligate DNA strands together. First, end-repair protein replaces lost nucleotides on the double-strand break and DNA ligase associates broken DNA fragments together. If fragments from different chromosomes ligate together, duplications or deletions of sequence can occur. **c** After the original stalling of the replication fork (*black line*), the lagging strand disengages and anneals to a second fork (*blue line*), followed by extension of the now ‘primed’ second fork and DNA synthesis. After the fork disengages, the tethered original fork with its lagging strand (*black and blue lines*) could invade a third fork (*green line*). Serial replication fork disengaging and lagging strand invasion could occur several times (e.g., FoSTeS × 2, FoSTeS × 3, etc.) before resumption of replication on the original template. It should be noted that the CNVs created through FoSTeS are difficult to be distinguished from those generated by micro-homology-mediated breakpoint-induced repair (MMBIR), a mechanism of end-joining that relies on small-scale homology of DNA sequence at the ends of DSBs

CNVs are very common and arise in the presence of specific architectural genomic elements that render DNA regions very susceptible to rearrangements. Depending on whether the same rearrangement is identified in unrelated individuals, CNVs can be grouped as recurrent or non-recurrent events (Lee and Lupski 2006). The most common cause of recurrent genomic rearrangements is the non-allelic homologous recombination (NAHR) that occurs between two DNA blocks of high homology, like the region-specific low-copy repeats sequences (LCRs) (Fig. 3a). On the contrary, non-recurrent CNVs can result from non-homologous end joining (NHEJ) or fork stalling and template switching (FoSTeS) mechanisms. NHEJ represents the major cellular mechanism for double-strand break repair: upon a double-strand break, NHEJ reconnects chromosome ends leaving random nucleotides at the site of the breakage to facilitate the strands’ alignment and ligation (Fig. 3b) (Ambroziak et al. 2015). FoSTeS occurs when the DNA replication machinery pauses, and the template is switched with another region in physical proximity to the original replication fork (Fig. 3c) (Lee et al. 2007). Such template switching may occur several times before the replication process gets back to its original template, resulting in complex rearrangements (Ambroziak et al. 2015).

CNVs can control phenotype in several ways: they can affect gene expression through the simple gene dosage effect, or through more intricate mechanisms such as insertions and deletions of regulatory regions and alterations of chromatin architecture (Gamazon and Stranger 2015). In this regard, CNVs can interfere with a form of regulatory scaffold of the chromatin (the so-called Topologically Associating Domains or TADs) by disrupting or repositioning boundaries and, therefore, constraining the enhancer or silencer activity with their target genes (Franke et al. 2016). Similarly, CNVs in other non-coding regions may alter the normal rate and tissue-specific transcription pattern of the neighboring, otherwise intact, genes by changing, for example, the affinity for transcription factors. This *cis*-*acting* effect of non-coding variations has been recently demonstrated for a SNP in a distal enhancer element regulating the expression of *SNCA* (Soldner et al. 2016). Some representative pictures about the mechanisms of non-coding variants and their implication in human genetics are reported in a number of excellent reviews (Spielmann and Mundlos 2016; Lupianez et al. 2016; Spielmann and Mundlos 2013), which the reader is referred to.

All together, CNV alterations may account for adaptive or behavioral traits, may have no phenotypic effects or can underlie diseases. For this reason, determining the clinical significance of CNVs is very challenging and comprehensively relies on frequency information from healthy control cohorts, hereditability, size, gene content, type (copy number state) and location on chromosome (interstitial, centromeric or repeat-regions) (Hehir-Kwa et al. 2013).

Notwithstanding the difficulties in interpreting quantitative data, specific large CNVs and single-gene dosage alterations have emerged as critical elements for the development and maintenance of the nervous system (Gu and Lupski 2008) and have appeared to contribute to hereditable or sporadic neurological diseases, such as neuropathies, epilepsy forms, autistic syndromes, psychiatric illnesses and also neurodegenerative diseases, including PD (Lee and Lupski 2006; Kalman and Vitale 2009; Hoyer et al. 2015; Olson et al. 2014; Grayton et al. 2012; Wang et al. 2013).

In the next paragraphs, we will focus on the current evidence about the occurrence of CNVs in familiar PD genes by highlighting strengths and weaknesses of interpretations for diagnosis and biomarkers usefulness. Moreover, we will collect from published literature the currently known set of rare CNVs observed in PD patients and analyze them through a *systems biology* point of view, in order to assess their biological role, their interactions and the possible functional impact on PD pathobiology.

## Copy number variations in familiar PD genes

### *SNCA*

*SNCA* (alpha-synuclein) represents the most convincing locus causing both familiar and sporadic PD. This gene encodes a small natively unfolded presynaptic protein that aggregates in Lewy bodies and Lewy neurites, the pathological hallmark lesions of PD (Stefanis 2012). As we will discuss here below, *SNCA* is the best example of dosage-dependent toxicity: the more alpha-synuclein you have, the worse will be PD.

The first genomic triplication of *SNCA* was observed within the Spellman–Muenter family (better known as Iowa Kindred), a large family with autosomal dominant inheritance transmission of PD and dementia (Singleton et al. 2003). Later, several families with different ethnic background have been described, including members carrying four copies (triplication) or three copies (duplication) of *SNCA* (Table 1) (Wang et al. 2013; Keyser et al. 2010; Sekine et al. 2010; Kojovic et al. 2012; Darvish et al. 2013; Olgiati et al. 2015; Ferese et al. 2015; Uchiyama et al. 2008; Ahn et al. 2008; Nishioka et al. 2006, 2009; Chartier-Harlin et al. 2004; Ibanez et al. 2004, 2009; Sironi et al. 2010; Pankratz et al. 2011; Elia et al. 2013; Konno et al. 2016; Ross et al. 2008; Kara et al. 2014; Mutez et al. 2011). In general, triplication generates very high expression of mRNA and protein molecules and influences the clinical manifestations of PD, causing severe forms of Parkinsonism similar to dementia with Lewy body. In contrast, the clinical phenotype of patients with duplicated *SNCA* resembles idiopathic PD, mainly with late age at onset, good efficacy for levodopa therapy, slower disease progression and without early development of dementia.

**Table 1:** All the current studies describing *SNCA* copy number changes in PD

CNVs in α-synuclein gene in PD						
CNV type	Size	Ethnicity	Phenotype	Methodology	F–S–D	References
Triplication (Spellman–Muenter family or Iowa Kindred)	1.61–2.04 Mb	Iowa	PD and dementia with LBs	qPCR, FISH	F	Singleton et al. (2003)
Duplication (Lister Family, branch J)	0.7987–0.9359 Mb	Sweden, United States	Late-onset parkinsonism and early dysautonomia	qPCR; Microsatellite markers analysis; Affymetrix 250 K microarray	F	Farrer et al. (2004), Fuchs et al. (2007)
Triplication (Lister Family, Swedish-America, Branch I)			Early-onset parkinsonism with dementia and dysautonomia			
Duplication (Ikeuchi family)	5 Mb	Japan	Progressive parkinsonism with dementia with LBs	Microsatellite markers analysis, qPCR	F	Ikeuchi et al. (2008)
Homozygous duplication (Ikeuchi family—consanguineous marriage)						
Duplication (Uchiyama family)	0.5–1.6 Mb	Japan	Parkinsonism with dementia with LBs	qPCR	F	Uchiyama et al. (2008)
Duplication	n.a.	Korea	Early-onset parkinsonism with rapidly progressive course, cognitive impairment, and dysautonomia (Ahn family)	Semi-quantitative multiplex PCR, FISH	F	Ahn et al. (2008)
			Typical PD		S	
Duplication	n.a.	Germany	Early-onset parkinsonism	qPCR, MLPA	D	Brueggemann et al. (2008)
Duplication	n.a.	European and North African	Early-onset PD	MLPA, Microsatellite markers analysis	S	Troiano et al. (2008)
Duplication (Family A)	0.6 Mb	Japan	Parkinsonism with or without dementia	Microsatellite markers analysis, qPCR, FISH, aCGH (BACS and Affymetrix)	F	Nishioka et al. (2009), Nishioka et al. (2006), Konno et al. (2016), Kara et al. (2014)
Duplication (Family B)	0.4 Mb					
Duplication (Family C)	0.4 Mb					
Duplication (Family D)	0.4 Mb					
Duplication (Family E)	0.2 Mb					
Duplication (Family F)	0.6 Mb					
Duplication (Family G)	0.6 Mb					
Triplication (FPD-014) (Pat 011)	2.61–2.64 Mb	France. Italy	Atypical autosomal dominant parkinsonism	Semi-quantitative Multiplex PCR, Microsatellite analysis, Affymetrix GeneChip Human Mapping 250 K microarray (just for P59 family: FISH, 44 k CGH arrays Agilent)	F	Chartier-Harlin et al. (2004), Ibanez et al. (2004), Ibanez et al. (2009), Ross et al. (2008), Kara et al. (2014), Mutez et al. (2011)
Duplication (FPD-131 o P59) (Pat 024-022-026)	4.928 Mb		Typical autosomal dominant PD			
Duplication (FPD-321) (Pat 021)	3.47–3.58 Mb					
Duplication (FPD-410) (Pat 001)	0.63–0.65 Mb					
Duplication (FPD-437) (Pat 010-012)	0.42–0.43 Mb					
Duplication (Sironi family)	3.65 Mb	Italy	PD with progression to dementia	MLPA, Agilent 105A chip	F	Sironi et al. (2010)
Duplication	n.a.	Belgium	Parkinsonian syndrome	Multiplex amplicon quantification, qPCR	S	Nuytemans et al. (2009)
Triplication (Keyser family)	n.a.	South African (French–Italian origin)	PD with dementia	MLPA, qPCR	F	Keyser et al. (2010)
Duplication	n.a.	Korea	PD with cognitive dysfunction	Semi-quantitative multiplex PCR	S	Shin et al. (2010)
Triplication	n.a.	Asian	Early-onset and severe clinical features of parkinsonism	qPCR, MLPA, microsatellite analysis	F	Sekine et al. (2010)
Duplication	3 Mb	n.a.	PD	Illumina370Duo arrays	F	Pankratz et al. (2011)
Homozygous duplication	0.928 Mb	Pakistan	Young-onset Parkinsonism	MLPA, Nimblegen 135 K-array CGH	F	Kojovic et al. (2012)
Duplication (Partial Trisomy 4q)	41.2 MB	Belgium	Young-onset, dopa-responsive parkinsonism	Karyotype, aCGH, MLPA	D	Garraux et al. (2012)
Duplication	n.a.	Non-Hispanic Caucasian	Autosomal dominant Early-onset PD	Customized 4 × 72 k format CGH microarrays by NimbleGen; Taqman qPCR	F	Wang et al. (2013)
Duplication (family Elia A)	773 Kb	Northern Argentina	Early-onset PD that was variably associated with nonmotor features, such as dysautonomia, cognitive deficits, and psychiatric disturbances	MLPA, qPCR, Affymetrix high-resolution single nucleotide polymorphism-array analysis	F	Elia et al. (2013)
Duplication (Family Elia B)	4820 Kb	Italian	Early-onset PD dementia with psychiatric disturbances to late-onset PD with mild cognitive impairment			
Duplication	6.4 Mb	Caucasian English	Atypical clinical presentation strongly reminiscent of frontotemporal dementia and late-onset pallidopyramidal syndromes	MLPA, aCGH Agilent 8 × 60 K	F	Kara et al. (2014)
Duplication	n.a.	Iranian	PD typical clinical features	MLPA, qPCR	F–S	Darvish et al. (2013)
Triplication			PD with dementia			
Duplication (mosaicism)	n.a.	American mitochondrial haplogroup and European autosomal markers	Early-onset Parkinsonism	MLPA (no dosage alteration in buccal swab), FISH (no rearrangements in peripheral leukocytes; duplication—triplication in oral mucosa)	F–S	Perandones et al. (2014)
Duplication	n.a.	American	Parkinsonism with LBs and Lewy neurites	n.a.	n.a.	Konno et al. (2016)
Triplication	1.3 Mb	Italian	Early-onset parkinsonism combined with depression, behavior disturbances, sleep disorders, and cognitive decline	Genome-wide SNP microarrays, FISH, MLPA	F	Olgiati et al. (2015)
Triplication	351 Kb	Italian	Severe parkinsonism featuring early-onset dyskinesia, psychiatric symptoms, and cognitive deterioration	CGH-Array, MLPA, qPCR	F	Ferese et al. (2015)

The CNVs mutation type, the size of the mutation, the ethnicity of patients, the phenotype and the methodological approaches to measure quantitative genomic variations are reported. The column F–S–D reports if described cases are familial, sporadic or de novo

An interesting familiar pedigree, the “Lister family”, presents both duplicated and triplicated *SNCA* carriers within different branches of the pedigree (branches J and I), suggesting a primary duplication event followed later by another one and resulting in the triplication (Farrer et al. 2004; Fuchs et al. 2007). Similarly, the Ikeuchi family has both heterozygous and homozygous duplication carriers born from a consanguineous marriage (producing a pseudo-triplication) (Ikeuchi et al. 2008). The clinical features of individuals with the *SNCA* homozygous duplication showed severe parkinsonism similar to that of triplication carriers.

Along with the familiar forms, a good percentage of sporadic PD patients carry de novo duplication of *SNCA* (Table 1) (Ahn et al. 2008; Garraux et al. 2012; Shin et al. 2010; Troiano et al. 2008; Brueggemann et al. 2008; Nuytemans et al. 2009). Generally, their clinical course is similar to typical sporadic PD without severe progression or cognitive decline.

The breakpoint of *SNCA* multiplications is not the same in each patient. The largest multiplication detected so far is about 41.2 Mb, containing 150 genes and defined a partial trisomy 4q (Garraux et al. 2012), while the smallest one counts about 0.2 Mb (Nishioka et al. 2009). The size and gene makeup of each multiplicated region do not seem to severely influence the clinical presentation of the carriers.

Interesting insights derive from the mosaicism condition of *SNCA* rearrangements. In this regard, two interesting PD cases have been described, which resulted negative to exon dosage test in peripheral blood, and positive for *SNCA* copy number changes on oral mucosa cells (Perandones et al. 2014). Both patients displayed a parkinsonian clinical phenotype of *SNCA* copy number carriers. Starting from this evidence, authors suggest to take into consideration the possibility to examine cells from both peripheral lymphocytes and other tissues to detect low-grade mosaicism.

### *PARK2*

Although *SNCA* story suggests a gain of function, several early-onset forms of PD have demonstrated the role of loss of function genes in the etiology of the disease. The most common loss-of-function mutations belong to Parkin (or *PARK2*) gene, one of the largest in our genome harbored in the long arm of chromosome 6 (6q25.2-q27) and encoding an E3 ubiquitin ligase. Mutations of *PARK2* are particularly frequent in individuals with familiar recessive inheritance and account for 50% of the cases with autosomal recessive juvenile PD. Parkin mutations also explain ~15% of the sporadic cases with onset before 45 (Bonifati 2012; Lucking et al. 2000) and act as susceptibility alleles for late-onset forms of PD (2% of cases) (Oliveira et al. 2003).

**Fig. 4:**
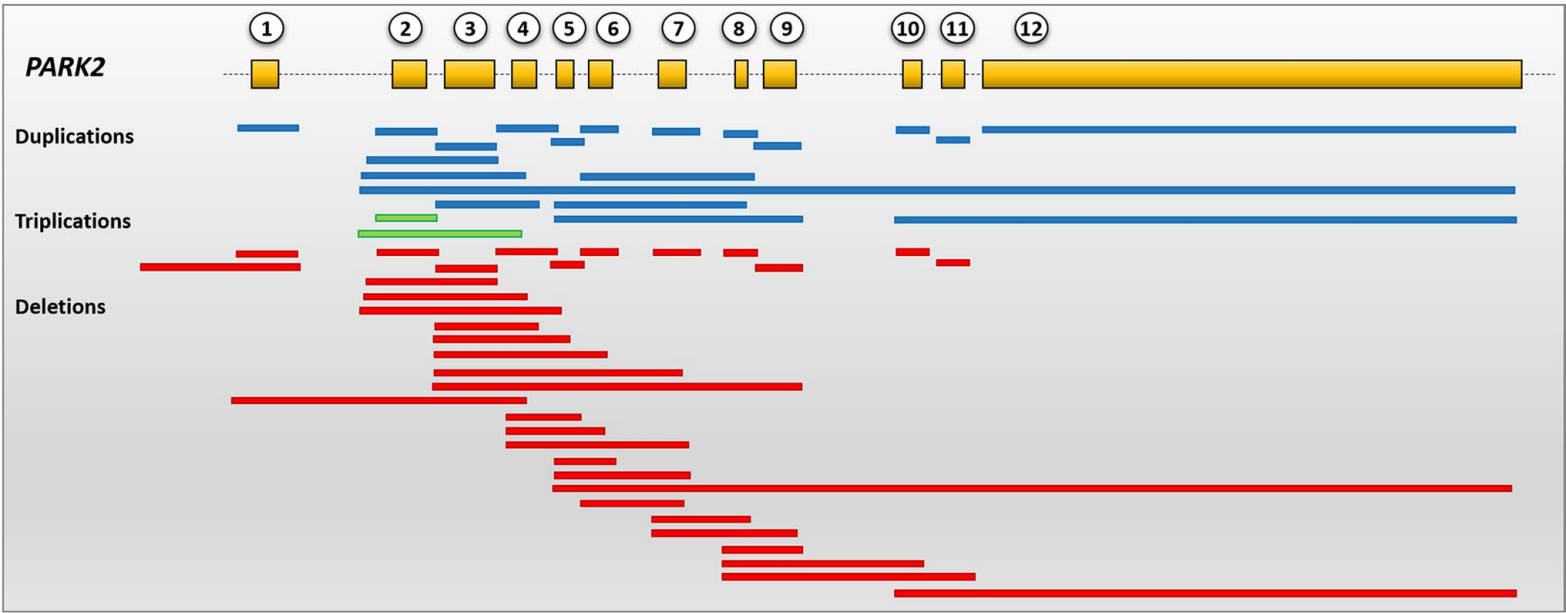
Schematic representation of *PARK2* genetic structure and currently identified CNVs in PD patients. All the canonical *PARK2* exons are involved in exons rearrangements. *Red bars* correspond to exons deletions, *blue bars* to duplications and *green bars* to triplications. All depicted CNVs can be found at the Parkinson Disease Mutation database (http://www.molgen.vib-ua.be/PDMutDB)

*PARK2* gene has a high mutation rate because it is located in the *core* of FRA6E site, one of the most mutation-susceptible common fragile sites of human genome (Ambroziak et al. 2015). For this reason, more than 200 putative pathogenic mutations have been reported so far, affecting numerous ethnic populations (Wang et al. 2010, 2013; Keyser et al. 2010; Nuytemans et al. 2009; Chaudhary et al. 2006; Klein et al. 2005; Shadrina et al. 2007; Choi et al. 2008; Pankratz et al. 2009; Kay et al. 2010; Guerrero Camacho et al. 2012; Moura et al. 2012; Yonova-Doing et al. 2012; Chu et al. 2014; Al-Mubarak et al. 2015; Guo et al. 2015; Mata et al. 2005). The *PARK2* mutation spectrum includes homozygous or compound heterozygous missense and nonsense point mutations, as well as several exon rearrangements (both duplications and deletions) involving all the originally cloned 12 exons and the promoter region. Recently, our research group has outlined a complex alternative splicing mechanism regulating the expression of *PARK2* (La Cognata et al. 2014, 2016; Scuderi et al. 2014). These data suggest that five additional exons exist, which, however, have never been considered for mutational or dosage screening. Overall, currently known Parkin CNVs are summarized in Fig. 4 and are collected in the Parkinson Disease Mutation database (http://www.molgen.vib-ua.be/PDMutDB), which the reader is referred to for more details.

CNV rearrangements involving *PARK2* exons account for 50–60% of all pathogenic anomalies, rendering gene dosage assays essential in parkin mutational screening (Kim et al. 2012). However, the *hotspot* nature of this gene makes its quantitative analysis a particular challenge, and several issues need to be pointed out in this regard. First, the determination of mutational phase of the rearrangements, meaning the assessment that amplified or deleted exons are really contiguous. Phase determination seems to be a fundamental requisite for *PARK2* molecular diagnosis: by phase determination, several patients with apparent contiguous multi-exon deletions were re-diagnosed as compound heterozygotes (Kim et al. 2012). A second important point refers to breakpoint mapping which can be useful to compare exon rearrangements between patients and families and to study the possible causing event mechanism (Elfferich et al. 2011). Just a few number of papers have addressed this issue so far, but mostly report rearrangements into the region between *PARK2* exons 2 and 8 (Ambroziak et al. 2015; Elfferich et al. 2011). In the majority of mapped cases, micro-homologies at breakpoint junctions were present, thus supporting NHEJ and FoSTeS as the major mechanisms responsible for *PARK2* genomic rearrangements (Ambroziak et al. 2015). Moreover, some data underpin the possible effects of ancient common founder in minor ethnic groups (Periquet et al. 2001). For example, microsatellite markers analysis in four families from The Netherlands have shown that a common haplotype of 1.2 Mb could be distinguished for the exon 7 duplication and a common haplotype of 6.3 Mb for the deletion of exon 4, suggesting common founder effects for distinct large rearrangements in parkin (Elfferich et al. 2011).

A relevant matter of ongoing debates is the pathogenic role of single heterozygous *PARK2* CNVs. Several studies have sought to address this issue, but the findings published so far are controversial and conflicting. Some reports indicate that CNVs heterozygous mutations in *PARK2* associate with increased PD risk (Pankratz et al. 2011; Pankratz et al. 2009; Huttenlocher et al. 2015), while others found no differences for association (Wang et al. 2013; Kay et al. 2010). In addition, examinations of family pedigrees revealed heterozygous members with mild late-onset PD (Klein et al. 2000; Farrer et al. 2001), or without typical clinical signs of the disease (Wang et al. 2013).

### *PINK1*

Pathogenic mutations in *PINK1* (PTEN-induced kinase gene) are a less common cause of early-onset PD with a frequency variable from 1 to 9% depending on the ethnic background (Pogson et al. 2011). The encoded protein is a putative serine/threonine kinase of 581 amino acids involved in mitochondrial quality control and oxidative stress (Valente et al. 2004).

Homozygous and compound heterozygous deletions involving different combinations of exons 4–8 have been described in both familial and sporadic early-onset cases coming from Japan, Brazil, Sudan and Iran (Table 2) (Darvish et al. 2013; Atsumi et al. 2006; Li et al. 2005; Camargos et al. 2009; Cazeneuve et al. 2009). A breakpoint analysis has been performed just in one of these patients, revealing a complex rearrangement involving the neighboring *DDOST* gene and maybe resulting from FoSTeS mechanism (Cazeneuve et al. 2009). Moreover, single heterozygous cases have been described, albeit these mutations do not completely explain the recessive inheritance pattern. The largest heterozygous deletion known so far (56 kb) includes the entire *PINK1* genetic region, two neighboring genes, and two highly similar AluJo repeat sequences, which have been suggested as responsible for an unequal crossing-over (Marongiu et al. 2007). Further heterozygous deletions involving exons 1, 3–8 and exon 7 have been described in familial or sporadic cases of early-onset PD (Table 2) (Moura et al. 2012; Guo et al. 2010; Samaranch et al. 2010).

**Table 2:** All the current studies describing *PINK1* copy number changes in PD

CNVs overlapping PINK1 gene in PD						
CNV type	Size	Ethnicity	Phenotype	Methodology	F–S	References
Homoz. deletion	Exons 6–8	Japan	Early-onset PD + dementia	n.a.	F	Li et al. (2005)
Homoz. deletion	~4600 bp	Japan	Early-onset PD	n.a.	n.a.	Atsumi et al. (2006)
Heteroz. deletion	~56 kb	Italy	Definite PD	qPCR, FISH, Microsatellite markers analysis	S	Marongiu et al. (2007)
Homoz. deletion	Exon 7	Brazil	Early-onset PD	Sequencing, qPCR	S	Camargos et al. (2009)
Homoz. deletion	8669 bp (exons 4–8)	Sudan	Early-onset PD	MLPA, sequencing	F	Cazeneuve et al. (2009)
Heteroz. deletion	Exons 3-8	China	Early-onset PD	qPCR	S	Guo et al. (2010)
Heteroz. deletion	Exon 7	Spanish	Early-onset PD with LBs	Sequencing, qPCR	F	Samaranch et al. (2010)
Compound heteroz. deletion	Exon 2 + exons 2–4	Iran	Typical clinical features	MLPA, qPCR	S	Darvish et al. (2013)
Homoz. deletion	Exon 5 and exon 4		Typical clinical features and PD with dementia		F	
Heteroz. deletion	Exon 1	Brazil	Early-onset PD	MLPA, qPCR	n.a.	Moura et al. (2012)

The CNVs mutation type, the size of the mutation, the ethnicity of patients, the phenotype and the methodological approaches to measure quantitative genomic variations are reported. The column F–S reports if described cases are familial or sporadic

### *PARK7*

*PARK7* was the third gene identified in 2001 as responsible of early-onset PD (Bonifati et al. 2003; van Duijn et al. 2001). It encodes a conserved multifunctional protein belonging to the peptidase C56 family (also called DJ1) which acts as a positive regulator of transcription, redox-sensitive chaperone, sensor for oxidative stress, and apparently protects neurons from ROS-induced apoptosis (Lev et al. 2006; Ariga et al. 2013; Xu et al. 2005).

The proof that *PARK7* was a gene-causing disease came from a study on a Dutch family where members carried a 14 kb homozygous deletion involving the first five of seven exons (Bonifati et al. 2003). Later, three siblings of Iranian origins born from consanguineous parents and carriers of a homozygous deletion of exon 5 have been reported (Table 3) (Darvish et al. 2013). Further heterozygous CNVs (both deletions and duplication) involving the exons of *DJ*-*1* gene have been published so far (Guo et al. 2010; Hedrich et al. 2004; Djarmati et al. 2004; Macedo et al. 2009), although they do not completely explain the recessive pattern of the PD phenotype.

**Table 3:** All the current studies describing *PARK7* copy number changes in PD

CNVs overlapping PARK7 gene in PD						
CNV type	Size	Ethnicity	Phenotype	Methodology	F vs. S	References
Homoz. deletion	Exons 1–5 (14.082 bp)	Dutch	Autosomal recessive early-onset Parkinsonism	Microsatellite markers analysis, cloning, PCR, sequencing	F	Bonifati et al. (2003)
Heteroz. deletion	Exons 5–7	Caucasian (Tyrol, Austria)	Early-onset PD	Quantitative duplex PCR	S	Hedrich et al. (2004)
Heteroz. deletion	Exon 5	Serbian	Early-onset PD	qPCR	n.a.	Djarmati et al. (2004)
Heteroz. duplication	Exons 1–5	Dutch	Early-onset PD	MLPA, sequencing	S	Macedo et al. (2009)
Heteroz. deletion	Exon 2	China	Early-onset PD	qPCR	S	Guo et al. (2010)
Homoz. deletion	Exon 5	Iran	Typical clinical features	MLPA, qPCR	F	Darvish et al. (2013)

The CNVs mutation type, the size of the mutation, the ethnicity of patients, the phenotype and the methodological approaches to measure quantitative genomic variations are reported. The column F–S reports if described cases are familial or sporadic

### *ATP13A2*

*ATP13A2* mutations are associated with Kufor-Rakeb syndrome (KRS), a form of recessively levodopa-responsive inherited atypical Parkinsonism (Vilarino-Guell et al. 2009). This gene encodes a large protein belonging to the ATPase transmembrane transporters, and recently it has been identified as a potent modifier of the toxicity induced by alpha-synuclein (Murphy et al. 2013). To our knowledge, just one family from Iran with deletion of *ATP13A2* has been reported, including three affected siblings born from consanguineous parents and carriers of a homozygous deletion of exon 2 (Darvish et al. 2013). All three individuals presented moderate mental retardation, aggressive behaviors, visual hallucinations, supranuclear vertical gaze paresis, slow vertical saccades and dystonia. Cognitive function deteriorated rapidly, and all of them developed dementia by age 10. Further clinical and genetic follow-up of KRS patients will increase the knowledge of the natural history and clinical features of this syndrome.

## The 22q11.2 deletion

A separate speech deserves the 22q11.2 deletion that lately is receiving more and more attention in PD field. Deletions at 22q11.2 are classically associated with a heterogeneous range of clinical syndromes, overall named 22q deletion syndrome (22qDS). The clinical phenotype of 22q deletion carriers varies widely, with multiple system involvement, including cleft palate, dysmorphic facial features, cardiac defects, skeletal deformities, developmental delays, learning disabilities and increased risk of developing schizophrenia and other mental disorders. Despite the multiple system involvement, the association between 22q11.2 deletion and PD was not suspected until the publication of independent case reports of co-occurrence of parkinsonism in patients with 22q11.2 deletion syndrome (Table 4) (Krahn et al. 1998; Zaleski et al. 2009; Booij et al. 2010).

**Table 4:** All the currently studies describing 22q11.2 deletions in PD patients

CNVs involving the 22q11.2 region						
CNV type	Size	Ethnicity	Phenotype	Methodology	Familial vs. sporadic	References
Heteroz. deletion	n.a.	n.a.	Childhood-onset schizophrenia associated with parkinsonism	FISH	No family history	Krahn et al. (1998)
Heteroz. deletion	3 Mb including COMT gene	n.a.	22qDS + early onset PD	FISH	No family history	Zaleski et al. (2009)
Heteroz. deletion	n.a.	n.a.	22qDS + PD (PD was considered a side effect of neuroleptic treatment or a clinical feature of early-onset PD)	FISH	n.a.	Booij et al. (2010)
Heteroz. deletion	From 15 to 3 Mb	Canada	22qDS + early-onset PD confirmed by neuropathological examination	FISH, quantitative real-time PCR	No family history	Butcher et al. (2013)
Mosaicism	n.a.	Ashkenazi Jewish	Midline defects and PD	FISH	Familial	Perandones et al. (2015)
Heteroz. deletion	2.88 Mb	n.a.	22q11.2DS + PD, dopa responsive	aCGH	Proband’s father developed PD in later life	Rehman et al. (2015)
Deletion	Eight patients, whose deletion ranged around 3 Mb	n.a.	Both early-onset and late-onset PD with typical motor signs and response to L-DOPA	Metanalysis of four previous independent studies and validation with CytoSure 15 K-Array	n.a.	Mok et al. (2016)

The CNVs mutation type, the size of the mutation, the ethnicity of patients, the phenotype and the methodological approaches to measure quantitative genomic variations are reported. The column familial vs. sporadic reports if described cases are familial or sporadic PD

The interest in this possible link increased after Butcher and colleagues reported four patients with early-onset PD in their study of 159 adults with 22q11.2 deletion syndrome, founding that the use of antipsychotics in these patients delayed diagnosis of PD, and assessing after autopsy examination the presence of typical Lewy bodies and Lewy neurite formations too (Butcher et al. 2013). A couple of months ago, Mok et al. (Mok et al. 2016) performed the reverse experiment, namely pooling data from previous large PD case–control studies and assessing the frequency of 22q11.2 deletion carriers. Eight patients with PD and none of the controls had the deletion, providing a statistical significant association between the 22q deletion and an increased risk of developing the disease (Table 4). In accordance with this result, a single case report from Virginia describes a 37-year-old early-onset PD patient carrying the 22q11.2 deletion but without any features of typical 22qDS (Rehman et al. 2015). All together, this evidence suggests 22q11.2 deletion might underlie early-onset PD, warning clinicians to take into consideration this genetic test as part of their evaluation for patients with early-onset PD.

The chromosome 22q11.2 region contains some excellent candidate genes for PD: *COMT* (or Catechol-O-Methyltransferase), a key regulator of synaptic dopamine levels and a target of inhibitory drugs for the treatment of wearing-off phenomena in PD patients (Muller 2015); *SEPT5*, a vesicle- and membrane-associated protein playing a significant role in inhibiting exocytosis, as well as a parkin substrate (Son et al. 2005; Marttinen et al. 2015); *DGCR8* that encodes a complex subunit involved in the biogenesis of microRNAs, including miR-185 which is predicted to target *LRRK2* (Ogaki and Ross 2014).

Interestingly, Perandones et al. (2015) reported a case of mosaicism of a patient from the Ashkenazi Jewish ethnic group with a history of midline defects and PD onset at 46 years (Table 4). In this patient, FISH test detected a mosaicism of the 22q deletion in 24% of the analyzed blood cells, highlighting the relevance of performing individual cell-by-cell analysis.

## High-throughput whole-genome studies to map CNVs in PD

The major reported PD-linked CNVs have actually been ascertained through single-gene investigations, and received most of the attention because of their already-known or hypothesized role in the disease. However, these mutations account only for a limited number of PD, and the vast majority of cases continue to remain without a valid explanation. Thanks to the rapid advancement of biotechnologies, scientists are now able to scan entirely the human genome, producing high-quality ultradense genotypes and fast localization of genomic deletions and duplications. However, their applications in PD field are still not numerous, and only a few studies have investigated the overall contribution of global CNVs on PD etiology (Pankratz et al. 2011; Simon-Sanchez et al. 2008; Liu et al. 2013; Pamphlett et al. 2012; Bademci et al. 2010).

The first pilot analysis assessing the role of structural genetic variations in risk for PD was carried out in a population of 276 unique and unrelated Caucasian individual with PD using two genome-wide SNP genotyping platforms and corrected metrics for CNVs interpretation (Simon-Sanchez et al. 2008). In this study, along with several *PARK2* deletions and duplications confirmed by independent gene dosage experiments, a total of 182 genomic duplications and 161 heterozygous/homozygous deletions were measured, but no statistically significant regions associated with PD were identified. Among these CNVs, a subgroup (38 duplications and 44 deletions) was revealed only in patients and not in healthy controls or in DGV repository (http://dgv.tcag.ca/dgv/app/home), a web database collecting CNV alterations observed in the normal population (Supplementary Table 1).

Some years later, Pankratz et al. (2011) presented the results of a systematic CNV genome-wide analysis performed using two CNV calling algorithms (PennCNV and QuantiSNP), two different association strategies (centric position and 400 kb window) and multiple filters to improve the quality of CNVs calls. By intersection of results from all these criteria, they were able to replicate the association of PD susceptibility with *PARK2* CNVs, and then revealed two novel genes (*DOCK5* and *USP32*) associated with an increase in risk for PD at genome-wide significance (unfortunately not confirmed by independent molecular tests). Also in this study, a set of altered genetic regions were unique of PD patients (Supplementary Table 1).

To identify novel CNVs and to evaluate their contribution to PD, Liu et al. (2013) conducted a CNVs genome-wide scan in a case–control dataset (268 PD cases and 178 controls), focusing on a genetic isolate, the Ashkenazi Jewish population. Using high-confidence CNVs, they examined the global genome-wide burden of large and rare CNVs: this analysis did not reveal significant differences between cases and controls, but deletions were found 1.4 times more often in cases than controls. Interestingly, several rare genic CNVs were present in patients and absent in controls (Supplementary Table 1). Among these, the duplication of *OVOS2* (ovostatin 2, a gene of unknown function) was classified as significant risk factors for PD. Other interesting PD-related CNVs alterations encompassed *NSF* and *WNT3* genes (later better discussed), and *ATXN3*, *FBXW7*, *CHCHD3*, *HSF1*, *KLC1*, and *MBD3*, which participate in the PD disease pathways.

An unusual approach was carried out by Pamphlett et al. (2012), who investigated the existence of somatic candidate genetic CNVs in PD brains and missing in blood DNA. A total of 45 PD-brain-specific CNVs was found, some of which overlap with DGV regions. Candidate genes (not in controls nor in DGV) included *BCL2* involved in mitochondrial function and apoptosis (discussed in the following paragraphs), *NRSN1* implicated in cellular vesicle formation, and *RYR2* which participates in cellular calcium release (Supplementary Table 1). This study shows that specific brain CNVs can be detected, and raises the possibility that brain-situated mutations could underlie some cases of PD.

## A *systems biology* approach for rare and singleton CNVs

**Fig. 5:**
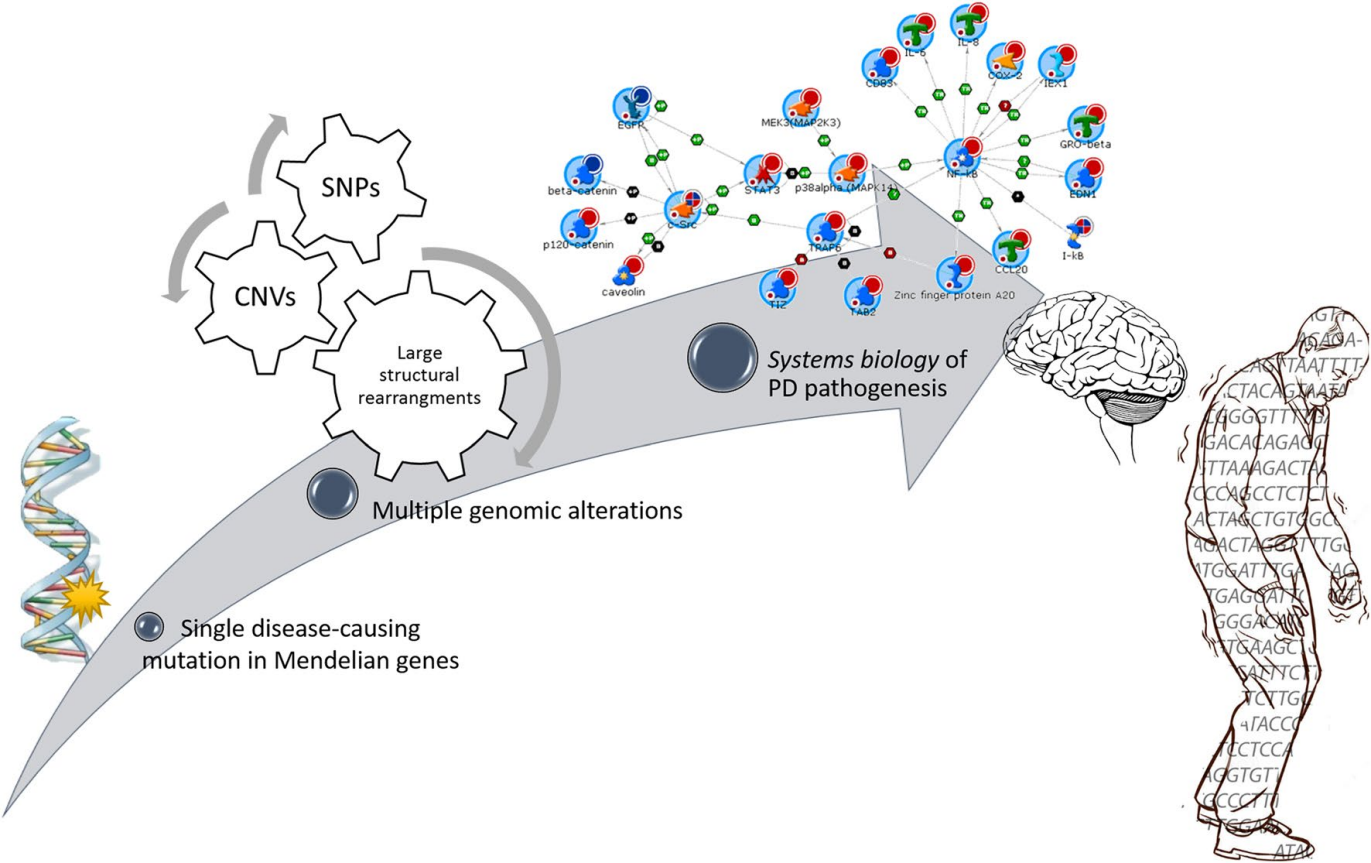
From a single-gene mutation perspective to a “*systems biology*” approach to dissect complex multifactorial diseases and improve the comprehension of the molecular basis underlying PD pathogenesis

Altogether, genome-wide studies have revealed the existence of multiple genetic loci containing rare o singleton copy number changes in PD and not reported in control cohorts (Supplementary Table 1). Although less frequent, these rare CNVs could represent potentially functional variants exerting small effects on PD pathogenesis, but not emphasized by single-gene investigations or association studies because do not reach a significant level of acceptance. These studies, in fact, are not the ideal approach for polygenic multifactorial diseases, where the pattern of allelic architecture could consist of hundreds of susceptibility loci acting together by modulating the disease itself. To overcome some of these limits, the *systems biology* perspective can be used to assess, in a comprehensive manner, the collective effect of these variants on PD outcome (Fig. 5).

**Fig. 6:**
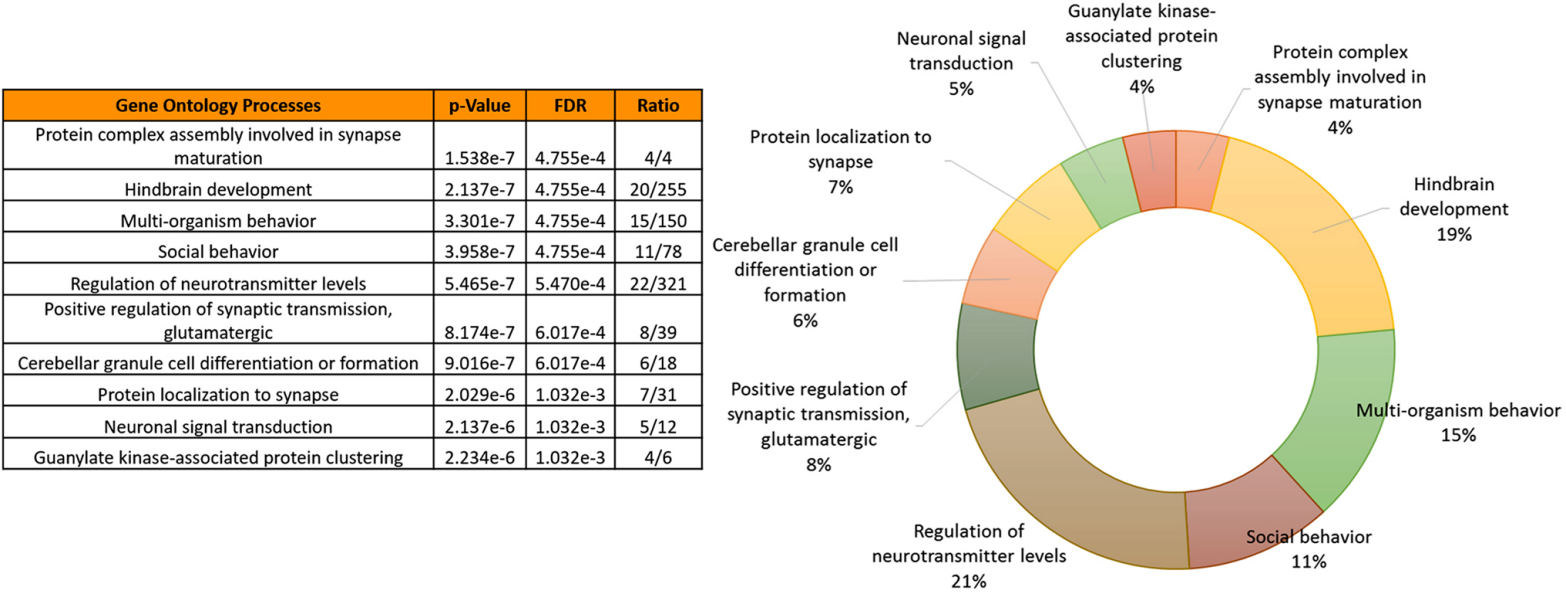
Gene ontologies (GO) enrichment analysis of PD-specific CNV loci reveals biological processes relevant for PD pathogenesis. **a** Representation of the top ten most significantly enriched (FDR < 0.05) canonical GO biological processes associated with candidate PD genes with copy number alterations (not reported in controls or DGV). The analysis was performed using MetaCore platform (GeneGo, Thompson Reuters). The list is arranged in descending order with the most significant biological process at the* top*. Detailed information about the entire list of CNVs and overlapping genes are reported in Supplementary Table 1. *p* values have been obtained through hypergeometric analysis and corrected by FDR (false discovery rate) method. **b** *Pie chart* representing the percentage of genes with altered copy number in PD belonging to the top ten enriched (*p* < 0.05) GO Biological Processes

**Fig. 7:**
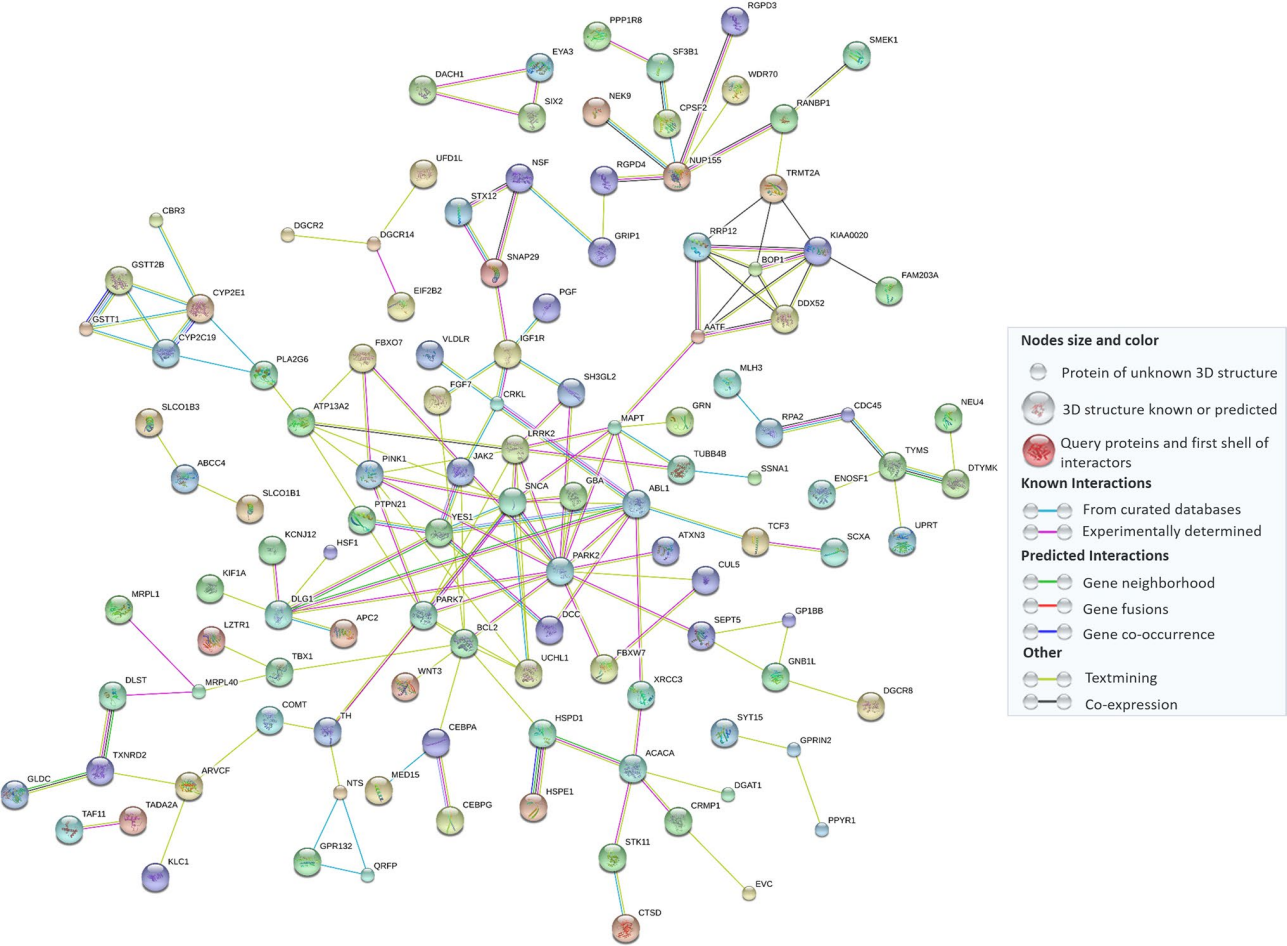
Potential protein–protein interactions among rare PD CNVs and the currently known Mendelian genes as shown by STRING Software v.10 (http://string-db.org/) with high confidence settings (0.700 as minimum interaction score). The *legend* displays the meaning of nodes and edges

Interestingly, the Gene Ontologies (GO) enrichment of the total CNV-driven genes observed in PD patients until now reveals common deregulated biological processes (Fig. 6) mainly related to nervous system functions and morphogenesis and including brain development (*p* value = 2.137E^−7^), regulation of neurotransmission (*p* value = 5.465E^−7^), neuronal signal transduction (*p* value = 2.137E^−6^) and social behavior (*p* value = 3.958E^−7^). Moreover, several potential relationships occur between rare CNV-affected genes and the currently known Mendelian PD genes (*SNCA*, *LRRK2*, *GBA*, *PARK2*, *PINK1*, *DJ1*, *VPS35, ATP13A2, PLA2G6, FBXO7, UCHL1, MAPT*). As shown in Fig. 7, specific and meaningful associations exist (i.e., proteins jointly contribute to a shared function, but this does not necessarily mean they are physically binding each other), and some rare CNV-affected genes could represent direct or indirect targets of Mendelian genes.

The global contribution of rare and singleton CNV-driven genes to nervous system pathophysiology and functions is also mirrored by the fact that, among those coinciding with the MIM MORBID/ORPHANET records, more than 50% are involved in syndromes with altered phenotypic nervous features, including ataxia conditions, neuropathies, dystrophies, learning and development disabilities and sensorineural disorders (Supplementary Table 2). All together, these findings support the evidence that uncommon individual CNVs may exert a susceptibility effect on PD, and strengthen the effectiveness of a *systems biology* approach to dissect complex multifactorial genetically heterogeneous diseases like PD.

**Fig. 8:**
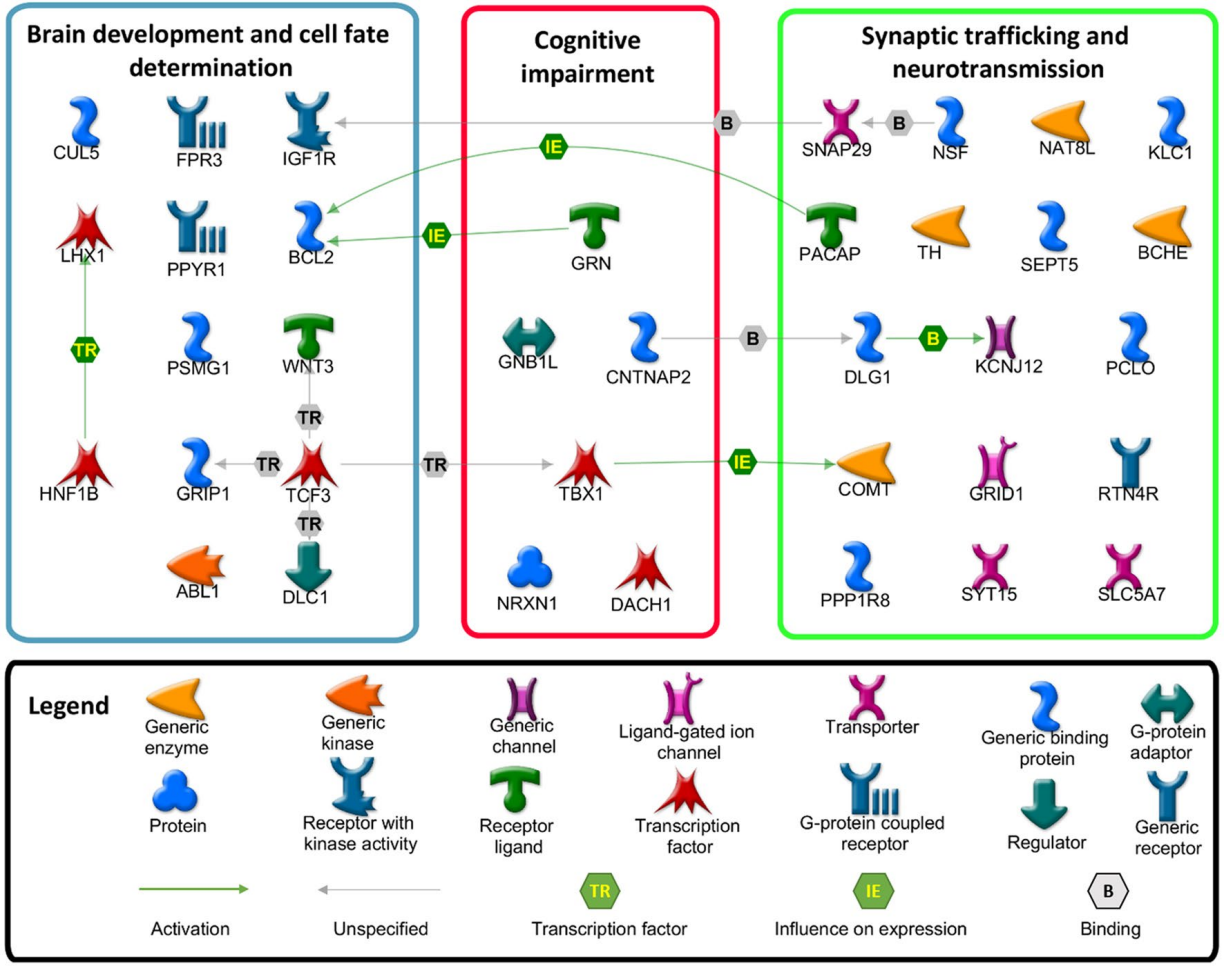
Interaction map representing CNV-altered genes enriched in GO classes and grouped on the basis of their main biological processes. The map was created using the MetaCore Pathway Map Creator tool (GeneGo, Thompson Reuters). Detailed information about type of CNV, chromosomal size and study references are reported in Supplementary Table 1

Below, we will briefly discuss the GO-enriched rare genes by grouping them into three main categories (Synaptic trafficking and neurotransmission, Brain development and cell fate differentiation, Cognitive impairment). GO-enriched genes are graphically illustrated in Fig. 8.

### Synaptic trafficking and neurotransmission

Synapses are specialized junctions of the central nervous system through which neurons connect each other to form extensive neural circuits. Synaptic functioning depends on a constant supply of energy and resources, essential for both neurotransmitters production and intracellular trafficking via repeated synaptic vesicle cycles. Alterations in synaptic stability result in a disruption of the neuronal networks, a common hallmark of several neurodegenerative conditions, including PD, Huntington’s and Alzheimer’s.

Along with the previously described *COMT*, some rare CNV-altered genes are involved in synaptic neurotransmission. One of the most interesting is the deletion of the entire *TH* (Tyrosine hydroxylase) gene, detected in a PD patient without evidence for dystonia but responsive to L-DOPA treatment, and in none of the controls (Supplementary Table 1) (Bademci et al. 2010, 2012). *TH* encodes a monooxygenase that catalyzes the conversion of l-tyrosine to L-dihydroxyphenylalanine (L-DOPA), the rate-limiting step in dopamine biosynthesis. Consistent with the essential role of TH in dopamine homeostasis, missense mutations in *TH* have been previously investigated, providing links with severe Parkinsonism-related phenotypes, such as Segawa’s syndrome, L-DOPA-responsive infantile Parkinsonism, or L-DOPA-responsive dystonia (DRD) in the recessive form (Bademci et al. 2012).

CNV-affected genes could alter not only dopamine metabolism but also other neurotransmitters’ signaling pathways. Convergent evidence agrees for early alterations in the cholinergic system in PD (Bohnen and Albin 2011; Muller and Bohnen 2013). In this regard, CNVs in the *BChE* gene, a nonspecific cholinesterase enzyme that hydrolyses many different choline-based esters, and in the synaptic choline transporter *SLC5A7* have been reported in some PD patients (Supplementary Table 1). Moreover, glutamate plays a central role in basal ganglia circuitry, sometimes modulated by dopamine itself. Genetic variations in the glutamate receptors *GRID1,* observed in some PD patients, could conceivably affect either the risk of developing PD or the phenotype.

*DLG1*, observed deleted in a PD patient, encodes a multi-domain scaffolding protein acting in septate junction formation, signal transduction, cell proliferation and synaptogenesis. Interestingly, this gene was identified as differentially expressed in the blood of PD patients vs. controls and was suggested at high confidence as candidate biomarker for PD (Sun et al. 2014). Moreover, recent studies have highlighted the role of *DLG1* in the regulation of 5-HT2AR endocytosis and signaling (Dunn et al. 2014).

Several genes working in the synaptic vesicular cargo trafficking are affected by CNV alterations, such as *SEPT5* and *SNAP29* (both overlapping the 22q11.2 deletion), *NSF, SYT15*, *PCLO,* and *KLC1*. A particular focus deserves *NSF* (also known as *N*-ethylmaleimide sensitive factor), previously identified as “top-hit” in a large GWAS meta-study (rs183211) (Liu et al. 2011). While *NSF* functions in vesicular trafficking, membrane fusion, and synaptic neurotransmission are well documented, some recent studies also suggest a direct interaction between NSF and the Dopamine D1 receptor (D1R) (Chen and Liu 2010). This gene was also experimentally showed to be the directed target of miR-4519, a microRNA whose genetic variants are strongly associated with PD (Ghanbari et al. 2016).

An interesting deletion concerns the *ADCYAP1* gene, encoding the pleiotropic bioactive peptide PACAP (or pituitary adenylate cyclase-activating polypeptide). PACAP is considered a potent neurotrophic and neuroprotective factor, playing an important role during the embryonic development of the nervous system, and protecting neurons against toxic insults and neurodegeneration (Reglodi et al. 2011; Lee and Seo 2014). In the specific case of PD pathology, PACAP has been demonstrated to safeguard in vitro PD cell model against both salsolinol-induced and inflammatory-mediated toxicity (Brown et al. 2013, 2014), to protect rat dopaminergic neurons after injection of 6-OHDA into the *substantia nigra* (Reglodi et al. 2004), and to prevent Parkinson-like neuronal loss and motor deficits induced by prostaglandin J2 (Shivers et al. 2014).

Further genes founded CNV-altered and involved in synaptic functions include ion channels (*KCNJ12*), biochemical enzymes (*NAT8L, PPP1R8*) and receptors (*RTN4R*).

### Brain development and cell fate determination

Brain development is an orchestrated, tightly regulated, and genetically programmed process with influences from the environment. Alterations in genes regulating differentiation, elaboration, and maintenance of neuronal cells can compromise neural specification events and cellular homeostasis, turning into neurodevelopmental abnormalities and neurodegenerative diseases (Mehler and Gokhan 2000).

Despite dopaminergic neurons cell death represents the leading event in PD pathology, it also physiologically occurs in developing brain during embryogenesis (van der Heide and Smidt 2013). The proper development of dopaminergic system requires the action of BCL2 family members, responsible for dictating cell survival or commitment to apoptosis (van der Heide and Smidt 2013). In this regard, a somatic deletion in *BCL2* has been observed in the brain of a PD patient in a homozygous state (Supplementary Table 1), likely producing deleterious effects on gene function.

Neuronal migration, differentiation, and death during brain development is also carefully tuned by a vast repertoire of growth and transcription factors, such as *LHX1* and *IGF1R,* observed duplicated in PD patients (Supplementary Table 1). *LHX1* encodes a transcription factor involved in axonal guidance and neurogenesis, and its overexpression has been demonstrated to inhibit the correct mesencephalic dopaminergic neurons differentiation (Nakatani et al. 2010). *IGF1R* produces the tyrosine kinase receptor for the IGF1 (insulin-like growth factor) signaling pathway. This pathway was found dysregulated in a previous cross-sectional transcriptomic analysis performed on PD datasets (Sutherland et al. 2009), and IGF1 signaling inhibitors have been proposed as promising therapies for the treatment of various late-onset neurodegenerative disorders (Cohen 2011). Moreover, the IGF1R reduction per se triggers protective effects in neurodegenerative conditions (Biondi et al. 2015), suggesting that its duplication could have a deleterious impact on neuronal life.

Members of the WNT family are additional factors expressed and secreted in the midbrain, involved in regulation of cell fate and patterning during embryogenesis. Among WNT family members, a singleton deletion in *WNT3* has been described in PD (Supplementary Table 1). *WNT3* is located near *MAPT* locus and, according to previous genome-wide association studies, variations in its genetic regions can influence the risk of developing PD (Liu et al. 2011; Simon-Sanchez et al. 2009). Moreover, the WNT/β-catenin signaling pathway is able to control the dopaminergic cell commitment in the midbrain, and is mediated by several transcription factors of TCF family (Chen 2013), such as *TCF3* observed deleted in PD (Supplementary Table 1).

Another interesting rare CNV-affected gene is *ABL1* (Supplementary Table 1), which encodes the tyrosine kinase protein c-Abl controlling neurogenesis, neurite outgrowth, and neuronal plasticity. Several lines of evidence suggest that aberrant activation of c-Abl plays an important role in PD pathogenesis: (1) c-Abl is upregulated in postmortem striatum of PD patients and its phosphorylation at Tyr412 is enhanced in substantia nigra and striatum; (2) c-Abl phosphorylates parkin and impairs its E3 ligase activity, leading to loss of dopaminergic neurons in SN; (3) c-Abl regulates the clearance of α-syn, catalyzing its phosphorylation mainly at Tyr39 and to a lesser extent at Tyr125; (4) the inhibition of c-Abl activity by several drugs (imatinib/Gleevec, nilotinib/Tasigna, bafetinib/INNO-406) protects against the loss of dopaminergic neurons in wild-type mice (Mahul-Mellier et al. 2014).

Additional CNV-altered genes, involved in brain development, neuronal migration, and synaptic plasticity, include G-coupled proteins (*FPR3, PPYR1*), transcription factors and coactivator (*HNF1B, GRIP1*), elements of ubiquitin/proteasome system (*CUL5, PSMG1*) and a member of the rhoGAP family (*DLC1*).

### Cognitive impairment

The core feature of cognitive decline associated with PD is represented by an impairment of executive functions. Deficits in planning, sequencing, and execution of complex-goal-directed behavior are usually reported; working memory, episodic memory, procedural learning and attention are compromised with the presence of attentive fluctuations (Calabresi et al. 2006).

A set of rare CNV-altered genes are involved in learning, behavior and cognitive dysfunctions, including *NRXN1*, *CNTNAP2*, *GRN*, *TBX1*, *GNB1L* and *DACH1*.

Neurexin 1, encoded by *NRXN1*, is a presynaptic neuronal adhesion molecule that interacts with postsynaptic neuroligins in both glutamatergic and GABAergic synapses, and is important in synaptic specification and efficient neurotransmission. Deletions and point mutations in *NRXN1* are associated with a broad spectrum of neuropsychiatric and neurodevelopmental disorders, including autism, intellectual disability, epilepsy, developmental delay, and schizophrenia (Jenkins et al. 2014). Like *NRXN1*, also *CNTNAP2* belongs to neurexin superfamily and encodes a neuronal transmembrane protein involved in neural-glia interactions and clustering of potassium channels in myelinated axons. Variations in this gene have been involved in susceptibility to neurodevelopmental disorders and language impairment (Infante et al. 2015). Moreover, downregulation of *CNTNAP2* has been associated with AD and PD conditions (Infante et al. 2015; van Abel et al. 2012). It has been suggested that neurexins–neuroligins level fluctuations sway the balance between excitatory and inhibitory neurotransmission, leading to damage of synapses and dendrites and maybe triggering protein aggregates in neurodegenerative conditions (Sindi et al. 2014).

*GRN* encodes progranulin, a multifunction protein widely distributed throughout the central nervous system primarily in neurons and microglia, and a potent autocrine neurotrophic factor and regulator of neuroinflammation (Van Kampen et al. 2014). Its loss-of-function mutations are known to be responsible for FTLDU-17 (ubiquitin-positive frontotemporal lobar degeneration linked to chromosome 17) and increase the risk for both Alzheimer’s and PD, suggesting important roles of progranulin in neurodegenerative processes (Chen et al. 2015). A deletion of *GRN* exons 1–11, resulting from a non-homologous recombination event, has been observed in a patient with typical GRN neuropathology, and in his sister presenting PD (Rovelet-Lecrux et al. 2008). Moreover, it has been recently demonstrated that progranulin gene delivery protects dopaminergic neurons in a PD model, suggesting that GRN gene therapy may have beneficial effects in the treatment of PD (Van Kampen et al. 2014).

Other CNV-driven genes include *TBX1* and *GNB1L,* both overlapping the 22q11.2 deletion, which have been associated with neuropsychiatric disorders such as schizophrenia and autism (Chen et al. 2012; Ishiguro et al. 2010; Hiramoto et al. 2011).

## Conclusions

A number of evidence suggests an extensive and complex genetic action of CNVs on PD etiopathogenesis. Thus far, unfortunately, only a small portion of the genetic variance has been identified; the remaining substantial components remain unknown and urgently need to be addressed. One way we can move on is using “*systems biology*”, a worthwhile instrument to analyze complex biological processes and generate a more definite molecular picture of PD.

In this review, we showed that disregarded individual rare CNVs functionally act in common deregulated biological processes relevant for PD pathogenesis and, therefore, potentially account for a portion of the “missing heritability” underlying PD. The comprehensive detection and functional characterization of rare CNVs in PD patients may be helpful to generate a more defined molecular picture of this complex disease, by revealing new candidate genes or disease-related molecular mechanisms, finally leading to improved diagnosis and counseling of mutation carriers. The forthcoming new era of genomics data promises to increase resolution and uncover new interesting clues.

## Electronic supplementary material

Below is the link to the electronic supplementary material.

Supplementary material 1 (XLSX 33 kb)

Supplementary material 2 (XLSX 15 kb)

## Abbreviations

*ABL1*: ABL proto-oncogene 1, non-receptor tyrosine kinase
*ADCYAP1*: Adenylate cyclase-activating polypeptide 1
*ATP13A2*: ATPase 13A2
*ATXN3*: Ataxin 3
*BCHE*: Butyrylcholinesterase
*BCL2*: Apoptosis regulator
*CHCHD3*: Coiled-coil-helix-coiled-coil-helix domain containing 3
*CNTNAP2*: Contactin-associated protein-like 2
CNVs: Copy number variations
*COMT*: Catechol-O-methyltransferase
*CUL5*: Cullin 5
D1R: Dopamine receptor D1
*DACH1*: Dachshund family transcription factor 1
*DDOST*: Dolichyl-diphosphooligosaccharide–protein glycosyltransferase non-catalytic subunit
*DGCR8*: DGCR8 microprocessor complex subunit
*DLC1*: DLC1 Rho GTPase-activating protein
*DLG1*: Discs large MAGUK scaffold protein 1
*DOCK5*: Dedicator of cytokinesis 5
*FBXO7*: F-box protein 7
*FBXW7*: F-box and WD repeat domain containing 7
FISH: Fluorescent in situ hybridization
*FoSTeS*: Fork stalling and template switching
*FPR3*: Formyl peptide receptor 3
FTLDU-17: Ubiquitin-positive frontotemporal lobar degeneration linked to chromosome 17
*GAK-DGKQ*: Cyclin G-associated kinase-diacylglycerol kinase theta
*GBA*: Glucosylceramidase beta
*GBAP1*: Glucosylceramidase beta pseudogene 1
*GNB1L*: G protein subunit beta 1 like
*GRID1*: Glutamate ionotropic receptor delta type subunit 1
*GRIP1*: Glutamate receptor interacting protein 1
*GRN*: Granulin
*HNF1B*: HNF1 homeobox B
*HSF1*: Heat-shock transcription factor 1
*HTRA2*: HtrA serine peptidase 2
IGF1: Insulin growth factor 1
*IGF1R*: Insulin-like growth factor 1 receptor
*KCNJ12*: Potassium voltage-gated channel subfamily J member 12
*KLC1*: Kinesin light chain 1
*LHX1*: LIM homeobox 1
*LRRK2*: Leucine-rich repeat kinase 2
*MAPT*: Microtubule-associated protein tau
*MBD3*: Methyl–CpG binding domain protein 3
NAHR: Non-allelic homologous recombination
*NAT8L*: *N*-Acetyltransferase 8 like
*NHEJ*: Non-homologous end joining
*NRSN1*: Neurensin 1
*NRXN1*: Neurexin 1
*NSF*: *N*-Ethylmaleimide-sensitive factor, vesicle fusing ATPase
*OVOS2*: Ovostatin 2
PACAP: Pituitary adenylate cyclase-activating polypeptide
*PARK10*: Parkinson disease 10
*PARK12*: Parkinson disease 12
*PARK16*: Parkinson disease 16
*PARK2*: Parkin RBR E3 ubiquitin protein ligase
*PARK3*: Parkinson disease 3 (autosomal dominant, Lewy body)
*PARK7*: Parkinsonism-associated deglycase
*PCLO*: Piccolo presynaptic cytomatrix protein
*PINK1*: PTEN-induced putative kinase 1
*PLA2G6*: Phospholipase A2 group VI
*PPP1R8*: Protein phosphatase 1 regulatory subunit 8
*PPYR1*: Neuropeptide Y receptor Y4
*PSMG1*: Proteasome assembly chaperone 1
*RTN4R*: Reticulon 4 receptor
*RYR2*: Ryanodine receptor 2
*SEPT5*: Septin 5
*SLC5A7*: Solute carrier family 5 member 7
*SNAP29*: Synaptosome-associated protein 29
*SNCA*: Alpha-synuclein
*SYNJ1*: Synaptojanin 1
*SYT15*: Synaptotagmin 15
*TBX1*: T-box 1
*TCF3*: Transcription factor 3
*TH*: Tyrosine hydroxylase
*UCHL1*: Ubiquitin C-terminal hydrolase L1
UPS: Ubiquitin proteasome system
*USP32*: Ubiquitin-specific peptidase 32
*VPS35*: VPS35 retromer complex component
*WNT3*: Wnt family member 3
5-HT2AR: Serotonin 2A receptor
